# Mass effect of a large frontal pneumosinus dilatans on the left frontal lobe: a case report and literature review

**DOI:** 10.1093/jscr/rjaf836

**Published:** 2025-10-26

**Authors:** Abdullah Alabdulqader

**Affiliations:** Department of Otolaryngology, Head and Neck Surgery, College of Medicine, Imam Mohammad Ibn Saud Islamic University, Uthman Ibn Affan Rd, An Nada, Riyadh 13317, Kingdom of Saudi Arabia

**Keywords:** pneumosinus dilatans, Dyke–Davidoff–Masson syndrome, frontal sinus, cerebral hemiatrophy

## Abstract

Pneumosinus dilatans (PD) is a rare condition characterized by abnormal expansion of the paranasal sinus without bone destruction or significant mucosal disease. Its association with Dyke–Davidoff Masson syndrome (DDMS) is particularly uncommon. We report the case of a 54-year-old female with learning disabilities, pseudoseizures, and asthma who was referred to our clinic after an MRI performed for decreased visual acuity revealed marked dilatation of the large left frontal sinus. She denied nasal symptoms but reported frontal headaches. Endoscopic examination was unremarkable. Imaging demonstrated progressive sinus enlargement and a mass effect in the left frontal lobe. The case was reviewed in a multidisciplinary setting and diagnosed as DDMS. Despite the conservative management, the patient remained stable. This case underscores the importance of recognizing PD and its rare associations, and emphasizes the value of multidisciplinary evaluation and radiological surveillance in selected patients.

## Introduction

Pneumosinus dilatans (PD) is an uncommon and poorly understood condition characterized by abnormal dilatation of one or more paranasal sinuses. The frontal sinus is most frequently involved, followed by the sphenoid, maxillary, and ethmoid sinuses. The etiology remains unclear, and the clinical presentation is variable, often complicating the diagnosis. Surgical treatment may be considered in cases with symptoms or complications [1–3].

Dyke-Davidoff-Masson Syndrome (DDMS) is a rare neurological disorder characterized by cerebral hemiatrophy, typically presenting with contralateral hemiparesis, seizures, and cognitive impairment. Diagnosis is established based on clinical features and characteristic radiological findings, particularly on magnetic resonance imaging (MRI) [4–6].

## Case presentation

A 54-year-old female with significant learning disabilities, pseudoseizures, and asthma was referred to our ENT clinic following an incidental finding of left frontal sinus expansion on MRI performed to evaluate bilateral visual deterioration. She denied nasal obstruction, rhinorrhea, or anosmia, but reported intermittent frontal headaches.

Endoscopic nasal examination revealed patent paranasal sinuses and healthy nasal mucosa. The patient was subsequently referred to the ophthalmology department for further evaluation and underwent computed tomography (CT) for detailed assessment of the sinuses. Medical management was initiated, consisting of saline nasal irrigation and topical nasal steroids (budesonide).

**Figure 1:**
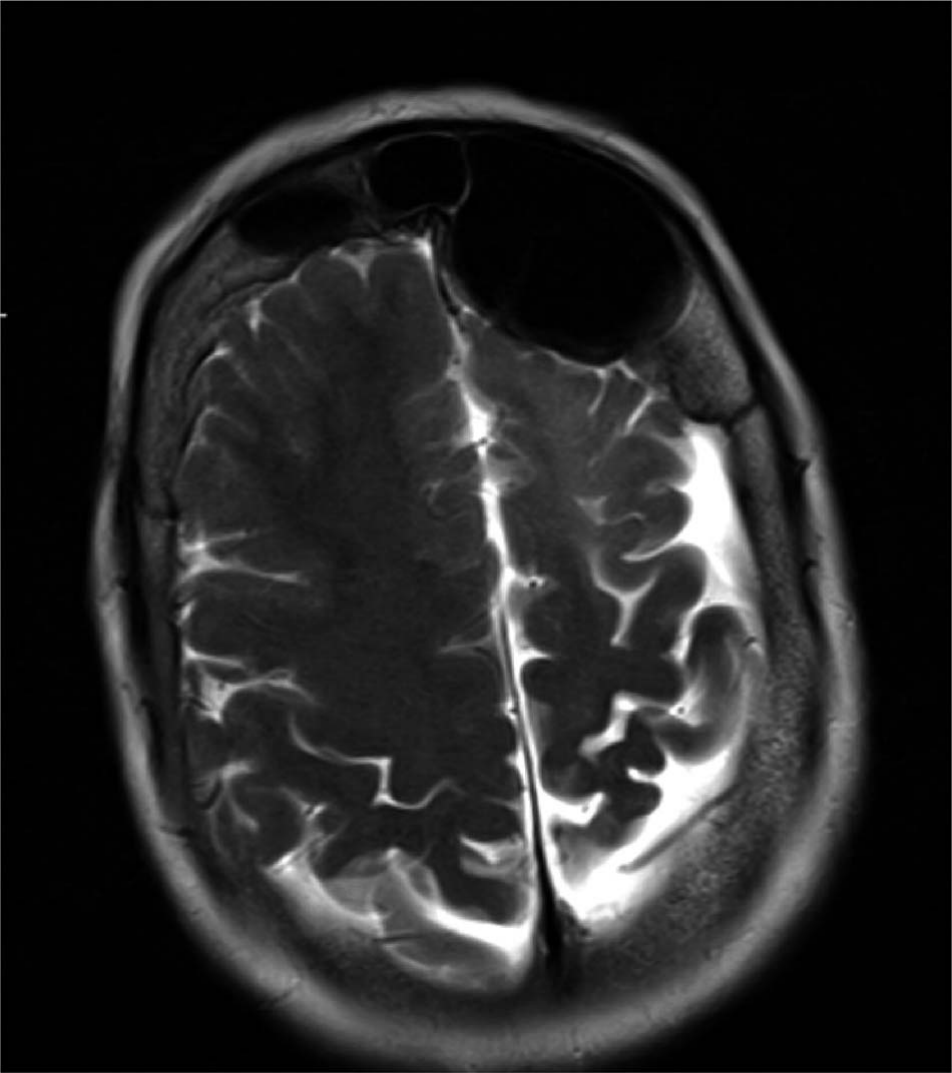
MRI showing enlarged left frontal sinus with mass effect on the frontal lobe.

**Figure 2:**
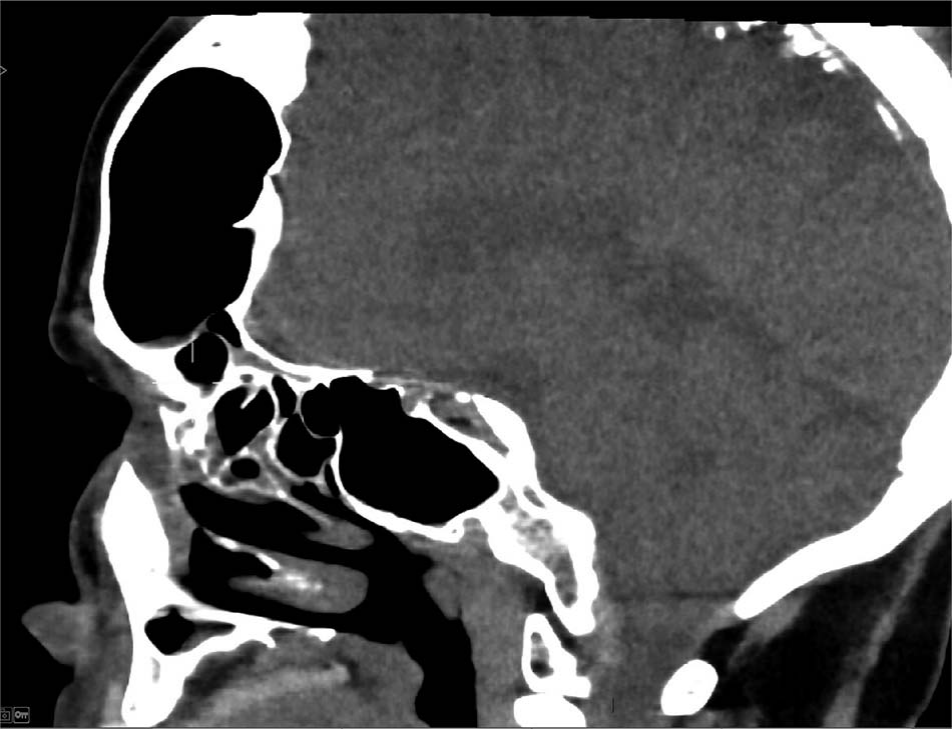
CT scan axial view showing hyperaeration of the left frontal sinus.

**Figure 3:**
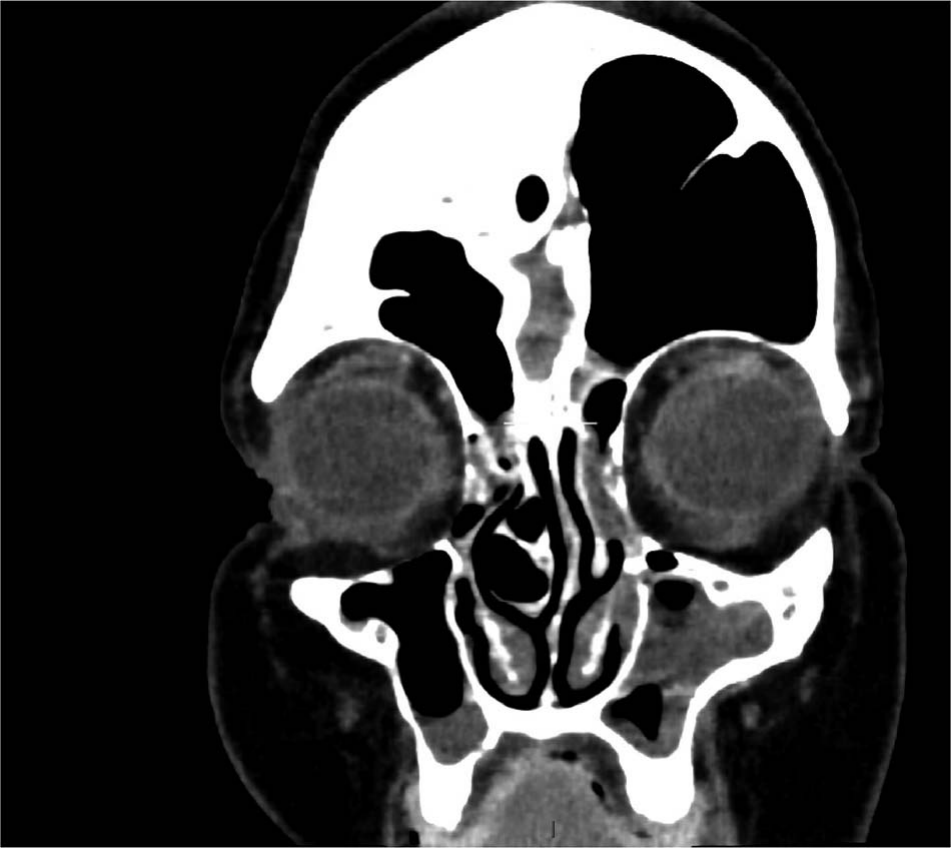
Coronal CT showing volume loss in the left hemisphere and enlarged sinus.

MRI demonstrated marked expansion of the left frontal sinus, consistent with PD, exerting a mass effect on the adjacent left frontal lobe and associated with a small area of increased T2-FLAIR signal intensity (Fig. 1). There was no evidence of compression of the lateral ventricle. However, mild prominence of the sulci and lateral ventricle on the left side suggested underlying parenchymal volume loss. No signs of hydrocephalus, midline shift, restricted diffusion, or susceptibility artifacts were identified. The optic nerve, optic chiasm, and optic tracts appeared normal. CT tomography confirmed these findings (Figs 2 and 3).

The imaging results were discussed with the patient and her sister, and the case was reviewed during a multidisciplinary skull base meeting. A presumptive diagnosis of DDMS was made, with associated left hemispheric dysplasia and cerebellar atrophy. Surgical intervention was deemed unnecessary in the absence of optic nerve involvement or visual compromise. A conservative management strategy with serial imaging follow-up was adopted.

At clinical follow-up, the patient remained stable, reporting only a mild left-sided headache. Ophthalmological assessment revealed preserved central visual pathways, with no significant visual deficits.

## Discussion

Frontal PD remains a poorly understood condition with proposed mechanisms including a ball-valve phenomenon, dysregulated bone remodeling, chronic low-grade infection with gas-forming bacteria, and congenital malformations [7]. Reported associations include frontoethmoidal meningocele, cognitive impairment, facial asymmetry, and various intracranial anomalies [8, 9].

DDMS is thought to result from early cerebral injury or developmental insult, leading to compensatory osseous changes such as calvarial thickening and hyperpneumatization of the frontal sinus [5]. In the present case, the marked enlargement of the frontal sinus likely represents a long-standing post-developmental compensatory process secondary to left hemispheric atrophy.

Neuroimaging is central to diagnosis. CT effectively delineates sinus morphology and bony alterations, while MRI is essential for identifying parenchymal abnormalities and excluding mass lesions [6, 10, 11].

Management should be individualized based on the patient’s symptoms. Most patients are managed conservatively. However, surgical intervention, including functional endoscopic sinus surgery or frontal sinus obliteration, may be considered in the presence of facial deformity, optic nerve compression, or refractory symptoms [3, 12, 13].

Although rare, PD should be considered in patients presenting with atypical sinus expansion and associated neurological symptoms. Its coexistence with DDMS is exceptionally uncommon and warrants a thorough imaging assessment and multidisciplinary evaluation. In the absence of progressive symptoms or complications, conservative management is an appropriate approach.
